# Leptin receptor is a key gene involved in the immunopathogenesis of thyroid‐associated ophthalmopathy

**DOI:** 10.1111/jcmm.16605

**Published:** 2021-05-14

**Authors:** Ziyi Chen, Zhe Chen, Jingya Wang, Meng Zhang, Xiaofei Wang, Deji Cuomu, Bingyin Shi, Yue Wang

**Affiliations:** ^1^ Department of Endocrinology The First Affiliated Hospital of Xi'an Jiaotong University Xi'an China; ^2^ Department of Spine Surgery Hong Hui Hospital Xi’an Jiaotong University Xi'an China; ^3^ Biomedical Experimental Center Xi'an Jiaotong University Xi'an China; ^4^ MOE Key Lab for Intelligent Networks & Networks Security School of Electronic and Information Engineering Xi'an Jiaotong University Xi'an China; ^5^ Genome institute The First Affiliated Hospital of Xi'an Jiaotong University Xi'an China; ^6^ Precision Medicine Center The First Affiliated Hospital of Xi'an Jiaotong University Xi'an China

**Keywords:** graves' disease, immune‐related genes, LEPR, leptin receptor, thyroid‐associated ophthalmopathy, weighted gene co‐expression network analysis

## Abstract

Thyroid‐associated ophthalmopathy (TAO), the most common and severe manifestation of Graves' disease (GD), is a disfiguring and potentially blinding autoimmune disease. The high relapse rate (up to 20%) and substantial side effects of glucocorticoid treatment further decrease the life quality of TAO patients. To develop novel therapies, we amid to explore the immunopathogenesis of TAO. To identify the key immune‐related genes (IRGs) in TAO, we integrated the IRG expression profiles in thyrocytes from a GD patient set (GD vs healthy control) and a TAO patient set (TAO vs GD). Gene Ontology (GO), Kyoto Encyclopedia of Genes and Genomes (KEGG), protein‐protein interaction (PPI) and receiver operating characteristic (ROC) curve analyses identified the leptin receptor (*LEPR*) gene as the key IRG in TAO immunopathogenesis. Gene set enrichment analysis (GSEA) suggested enrichment of the antigen presentation pathway in TAO patients with higher *LEPR*. Increased *LEPR* expression was validated in TAO orbital tissues, and weighted gene co‐expression network analysis (WGCNA) showed that cell adhesion processes were positively correlated with *LEPR*. Our study revealed that *LEPR* is a key gene in TAO immunopathogenesis and plays different roles in thyrocytes and orbital tissues. Our findings provide new insights into diagnostic and therapeutic biomarkers for TAO.

## 1 INTRODUCTION

Graves' disease (GD) is a common autoimmune thyroid disease caused by the breakthrough of tolerance to the thyroid‐stimulating hormone receptor (TSHR). With an annual incidence of approximately 20 to 50 cases per 100 000 persons,^1^ GD is associated with several complications, the most frequent and severe one of which is thyroid‐associated ophthalmopathy (TAO).^2^ Simultaneously or within 18 months of developing GD, almost half of GD patients develop TAO, whose symptoms include a dry and gritty ocular sensation, photophobia and excessive tearing.^3^ Signs of TAO are swollen extraocular muscles, expansion of orbital fat and connective tissue and, in severe cases, corneal ulceration and decreased visual acuity.^4^ Although glucocorticoids are a preferred first‐line treatment for TAO patients, approximately 20%‐30% of patients do not respond to glucocorticoids, and 10%‐20% experience relapse after treatment withdrawal.^3^, ^5^ Considering the unsatisfactory therapeutic effects and decreased quality of life associated with TAO, the development of novel therapies targeting its pathogenic mechanisms has always been recognized as imperative.^6^

The pathogenesis of TAO is still unclear but appears to involve both adaptive and innate immune responses. Regarding adaptive immune responses, single‐nucleotide polymorphisms (SNPs) in the *IL‐21* and *CTLA4* genes have been associated with TAO.^7^, ^8^ Both CD4+ and CD8+ T cells, as well as B cells, were found to be present in the majority of examined orbits from patients with TAO, and a 2018 study suggested that the level of infiltration correlates with disease activity.^9^ Additionally, orbital fibroblasts, considered key target cells in TAO, exhibit up‐regulation of CD40, which causes their activation by CD40L on T cells; moreover, TSHR‐expressing T cells might further stimulate adipogenesis via cyclooxygenase up‐regulation.^10^, ^11^ Regarding innate immune responses, macrophage infiltration in orbital fat from patients with TAO is higher than that in controls,^12^ possibly promoting a profibrotic phenotype in orbital fibroblasts through increased hyaluronic acid production and cell contractility.^13^ Based on these previous observations regarding the immune mechanism of TAO, advanced treatments, such as mycophenolate mofetil (an inhibitor of T and B cell proliferation), rituximab (an anti‐CD20 monoclonal antibody) and tocilizumab (an anti‐IL6 receptor antibody), have been proposed.^14^ However, these treatments have certain side effects. Thus, exploring the underlying pathogenesis and further developing novel therapies for TAO from the perspective of immune responses is a great need.

In this study, we first identified that the leptin receptor (*LEPR*) gene is the key gene involved in the immunopathogenesis of TAO *LEPR* expression was validated not only in thyrocytes but also in orbital tissues from TAO patients. Bioinformatic analyses revealed that up‐regulation of *LEPR* promotes antigen presentation in thyrocytes and mediates protein secretion, immune cell adhesion and adipogenesis pathway activity in orbital tissues. Our findings suggest that *LEPR* contributes to the immunopathogenesis of TAO and is a promising diagnostic and therapeutic target.

## 2 MATERIALS AND METHODS

### 2.1 Study design and data collection

**FIGURE 1:**
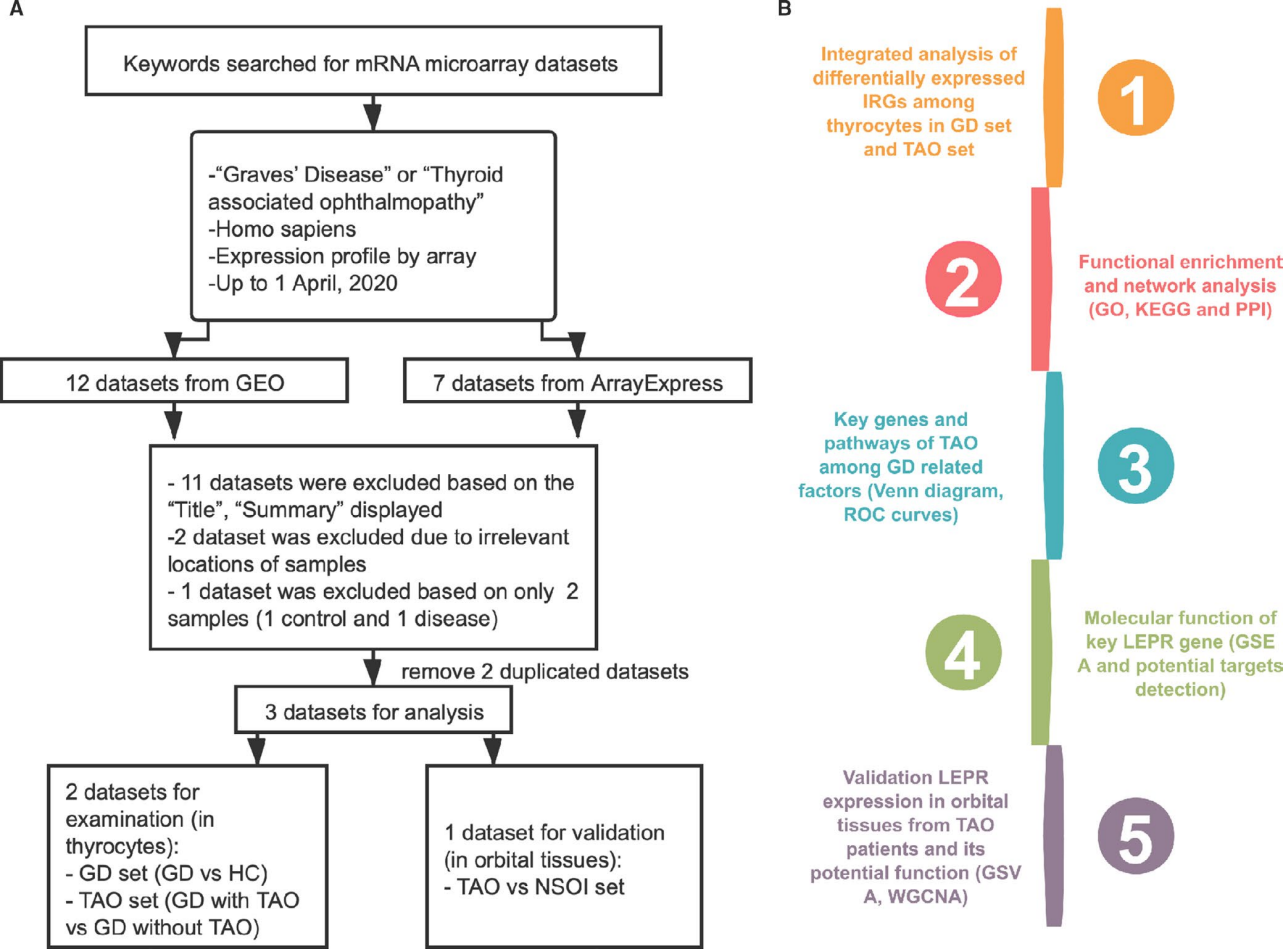
Data set selection flow chart and analysis processes. (A) Totally, 12 data sets from Gene Expression Omnibus (GEO) and 7 data sets from ArrayExpress were evaluated. Finally, 4 data sets for mRNAs were selected for the subsequent analysis. (B) The current study consisted of 5 steps, and the results of analysis were validated in other data sets

Three microarray data sets (E‐MEXP‐2612, GSE9340 and GSE58331) of GD or TAO patients were obtained from the NCBI Gene Expression Omnibus (GEO) and ArrayExpress in accordance with our selection criteria until 1 April 2020 (Figure 1). E‐MEXP‐2612 was used to analyse gene expression profiles of GD in thyrocytes (2 healthy controls [HCs] and 6 patients with GD), and GSE9340 was used to analyse thyrocytes from TAO patients (8 GD patients without TAO and 10 GD patients with TAO). The GSE58331 data set was used to validate the findings regarding thyrocytes in orbital tissues from TAO patients (28 patients with non‐specific orbital inflammation (NSOI), 24 patients with TAO and 21 HCs). The E‐MEXP‐2612, GSE9340 and GSE58331 data sets were generated utilizing the GPL571, GPL6014 and GPL570 platforms, respectively. The normalization and quality control of data in E‐MEXP‐2612 were carried out with the ‘affy’ R package (version 1.64.0),^15^ and the remaining data sets were processed with the ‘limma’ R package (version 3.42.2).^16^ In addition, a list of immune‐related genes (IRGs), which were identified to participate in immune activity, was obtained from the Immunology Database and Analysis Portal (ImmPort) database.^17^

### 2.2 Screening of differentially expressed genes and principal component analysis

Data analysis was conducted with the ‘limma’ R package to detect differentially expressed genes (DEGs) between the GD and HC data or the TAO and GD data after normalization. The following cut‐off criteria were used: |log_2_(fold change; FC)| > 0.1 and *P* value <.05. Next, the differentially expressed IRGs were extracted from the complete set of DEGs. Two features were extracted from the differentially expressed IRGs or complete gene set of each group via an unsupervised principal component analysis (PCA) method. Heatmaps and volcano plots were generated using the ‘pheatmap’ (version 1.0.12) and ‘ggplot2’ (version 3.3.0) packages.

### 2.3 Gene Ontology (GO) functional enrichment analysis, Kyoto Encyclopedia of Genes and Genomes (KEGG) pathway analysis and protein‐protein interaction (PPI) network construction

To explore the underlying mechanism by which differentially expressed IRGs impact the progression of GD and TAO, we conducted functional enrichment analyses, including GO term and KEGG pathway analyses, with the ‘clusterProfiler’ R package (version 3.14.3) ^18^ and set a *P* value of <.05 as the threshold. Biological processes (BP), cellular components (CC) and molecular functions (MF) were included in the GO term enrichment analysis. The R package ‘GOplot’ (version 1.0.2) ^19^ was adapted to graphically show the results of GO analyses. The PPI network was constructed based on data collected from the STRING online database (https://string‐db.org/; with a confidence score of >0.4 set as the cut‐off value) and visualized with Cytoscape software version 3.8.0^20^ (with genes with a degree of ≥3 considered key genes). In this part, the shared IRGs in the above analyses served as common key genes.

### 2.4 Identification of key genes and common pathways

The ‘VennDiagram’ R package (version 1.6.20)^21^ was used to conduct Venn diagram analysis for differentially expressed IRGs and pathways, which were obtained from data sets via GO, KEGG and PPI analyses. The diagrams show the shared IRGs (key genes in TAO) together with processes and pathways.

### 2.5 Evaluation of diagnostic efficacy

The Pearson correlation test was used to compare the expression of key genes with disease states. A receiver operating characteristic (ROC) curve was plotted, and the area under the curve (AUC) was calculated with the ‘pROC’ R package (version 1.16.2)^22^ to evaluate the capability of key genes to distinguish TAO from GD or TAO from NSOI. ROC curves were considered significant with AUC value >0.8.

### 2.6 Gene set enrichment analysis and gene set variation analysis

To further explore the potential function of the *LEPR* gene in TAO, gene set enrichment analysis (GSEA) and gene set variation analysis (GSVA) were performed for a single key gene. Patients in the data sets GSE9340 and GSE58331 were divided into two groups, according to the median expression of *LEPR*. The R packages ‘clusterProfiler’ and ‘GSVA’ (version 1.34.0) ^18^, ^23^ were used to perform GSEA and GSVA, respectively. The gene sets ‘c2.all.v6.2.symbols.gmt’, ‘c5.all.v6.2.symbols.gmt’ and ‘h.all.v6.2.symbols.gmt’ in the Molecular Signatures Database (MSigDB)^24^ were selected as the reference gene set, and a *P* value of <.01 was considered the threshold.

### 2.7 Molecular characteristics of the *LEPR* gene

To explore the regulatory mechanism of *LEPR* in GD and TAO, we focused on the genes in the leptin signalling pathway that may be targets for *LEPR*. The gene list was obtained from the Reactome Pathway Database (https://reactome.org). The expression profiles of these genes were visualized using the ‘forestplot’ R package (version 1.9). The genes that were differentially expressed and significantly correlated with *LEPR* expression were considered potential target genes for *LEPR* in the TAO set.

### 2.8 Weighted gene co‐expression network analysis

Gene co‐expression network analysis was specifically performed on TAO patient data in the GSE58331 data set using the R package weighted gene co‐expression network analysis (WGCNA; version 1.69).^25^ The similarity matrix was constructed by Pearson correlation analysis. Next, to achieve a scale‐free topology (scale‐free *R* ^2^ > .85), an appropriate soft‐thresholding power β was chosen with the function ‘pickSoftThreshold’ in the ‘WGCNA’ package. The transformed expression matrix was used as input into the WGCNA package, and Functions, modules and corresponding eigengenes were obtained. The interactions (correlations) of each module with *LEPR* expression were analysed and visualized in a heatmap. For modules that were significantly correlated with *LEPR* expression (*P* value <.05), GO analysis was conducted via the ‘clusterProfiler’ R package.^18^

### 2.9 Statistical analysis

The statistical significance of differences between two groups was analysed by a non‐parametric test or *t* test based on the data distribution characteristics. All analyses were performed with R3.6.2 software. A *P* value of <.05 was considered statistically significant.

## 3 RESULTS

### 3.1 Identification of differentially expressed IRGs in the GD set

**FIGURE 2:**
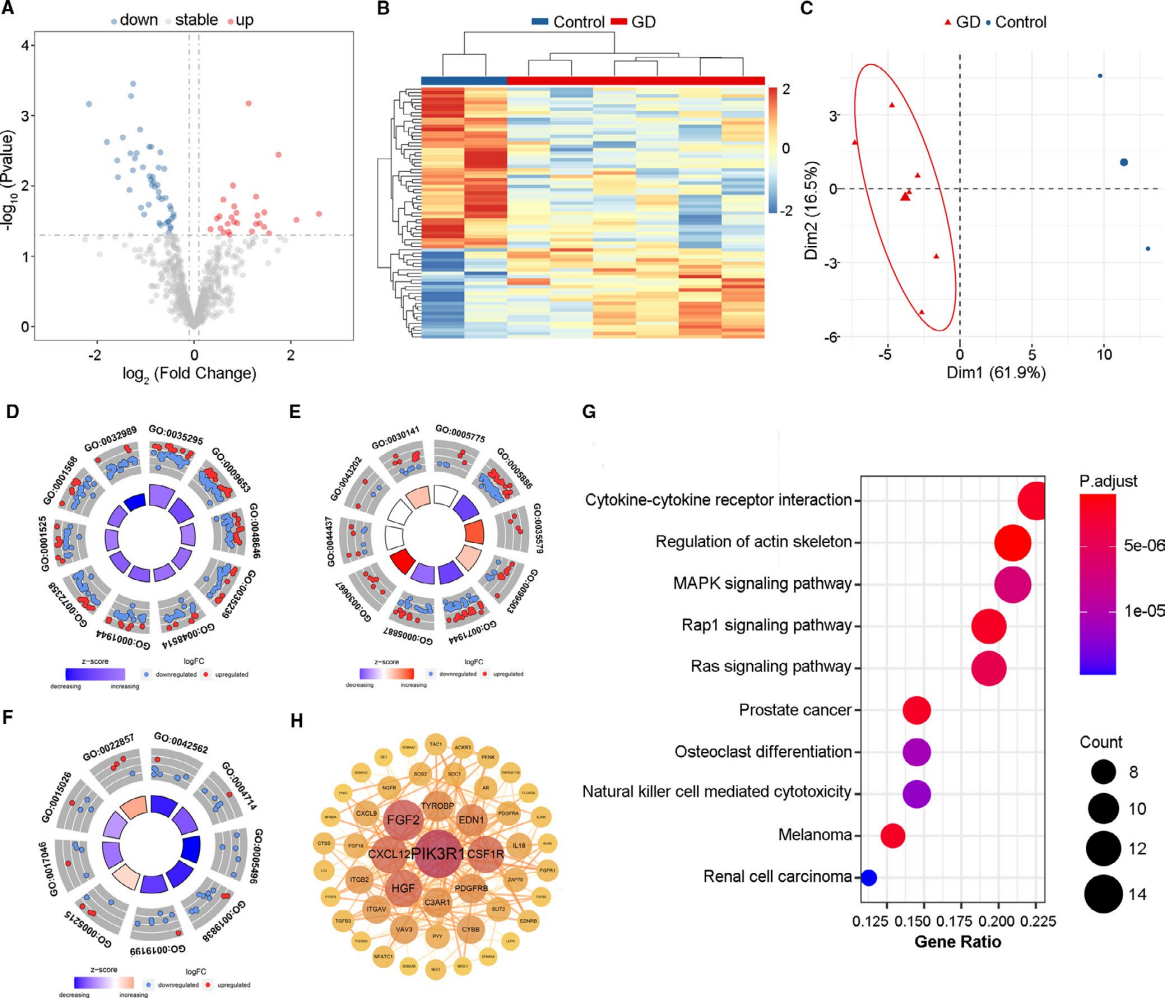
Identification of differentially expressed immune‐related genes (IRGs) in GD vs HC set. (A) Volcano plot showed differentially expressed IRGs between GD and HC group. Blue dots represented down‐regulated genes and red dots were up‐regulated genes. (B) Heatmap and (C) PCA plot showed obvious differences with those differentially expressed IRGs in GD and HC group. (D) Gene Ontology enrichment analysis of differentially expressed IRGs among biological processes, (E) cellular components and (F) molecular functions. (D) GO:0035295 tube development; GO:0009653 anatomical structure morphogenesis; GO:0048646 anatomical structure formation involved in morphogenesis; GO:0035239 tube morphogenesis; GO:00485114 blood vessel morphogenesis; GO:0072358 cardiovascular system development; GO:0001525 angiogenesis; GO:0001568 blood vessel development; GO:0032989 cellular component morphogenesis. (E) GO:0005775 vacuolar lumen; GO:0005886 plasma membrane; GO:0035579 specific granule membrane; GO:0099503 secretory vesicle; GO:0071944 cell periphery; GO:0005887 integral component of plasma membrane; GO:0030667 secretory granule membrane; GO:0044437 vacuolar part; GO:0043202 lysosomal lumen; GO:0030141 secretory granule. (F) GO:0042562 hormone binding; GO:0004714 transmembrane receptor protein tyrosine kinase activity; GO:0005496 steroid binding; GO:0019838 growth factor binding; GO:0019199 transmembrane receptor protein kinase activity; GO:0005215 transporter activity; GO:0017046 peptide hormone binding; GO:0015026 coreceptor activity; GO:0022857 transmembrane transporter activity. (G) The top 10 most significant Kyoto Encyclopedia of Genes and Genomes pathways. (H) Protein‐protein interaction network of potential key IRGs. GD, Graves’ disease; HC, healthy control; PCA, principal component analysis

We identified 298 up‐regulated and 897 down‐regulated genes in the GD set in the E‐MEXP‐2612 data set (thyrocytes; GD vs HC; Figure S1A). From this set of genes, we extracted 75 differentially expressed IRGs,^17^ specifically, 27 up‐regulated and 48 down‐regulated genes (Figure 2A). The heatmap showed that the differentially expressed IRGs could discriminate the GD and HC groups (Figure 2B), and the PCA plot of the GD group did not overlap with the profile of the HC group (Figure 2C). Gene functional enrichment analysis revealed that ‘tube development’, ‘plasma membrane’ and ‘hormone binding’ were the most enriched biological terms (Figure 2D,E). The KEGG pathway cytokine‐cytokine receptor interaction was the most highly enriched with differentially expressed IRGs (Figure 2F). The PPI network of differentially expressed IRGs was constructed, and 50 genes were identified as potential key IRGs in GD (Figure 2G).

### 3.2 Identification of differentially expressed IRGs in the TAO set

**FIGURE 3:**
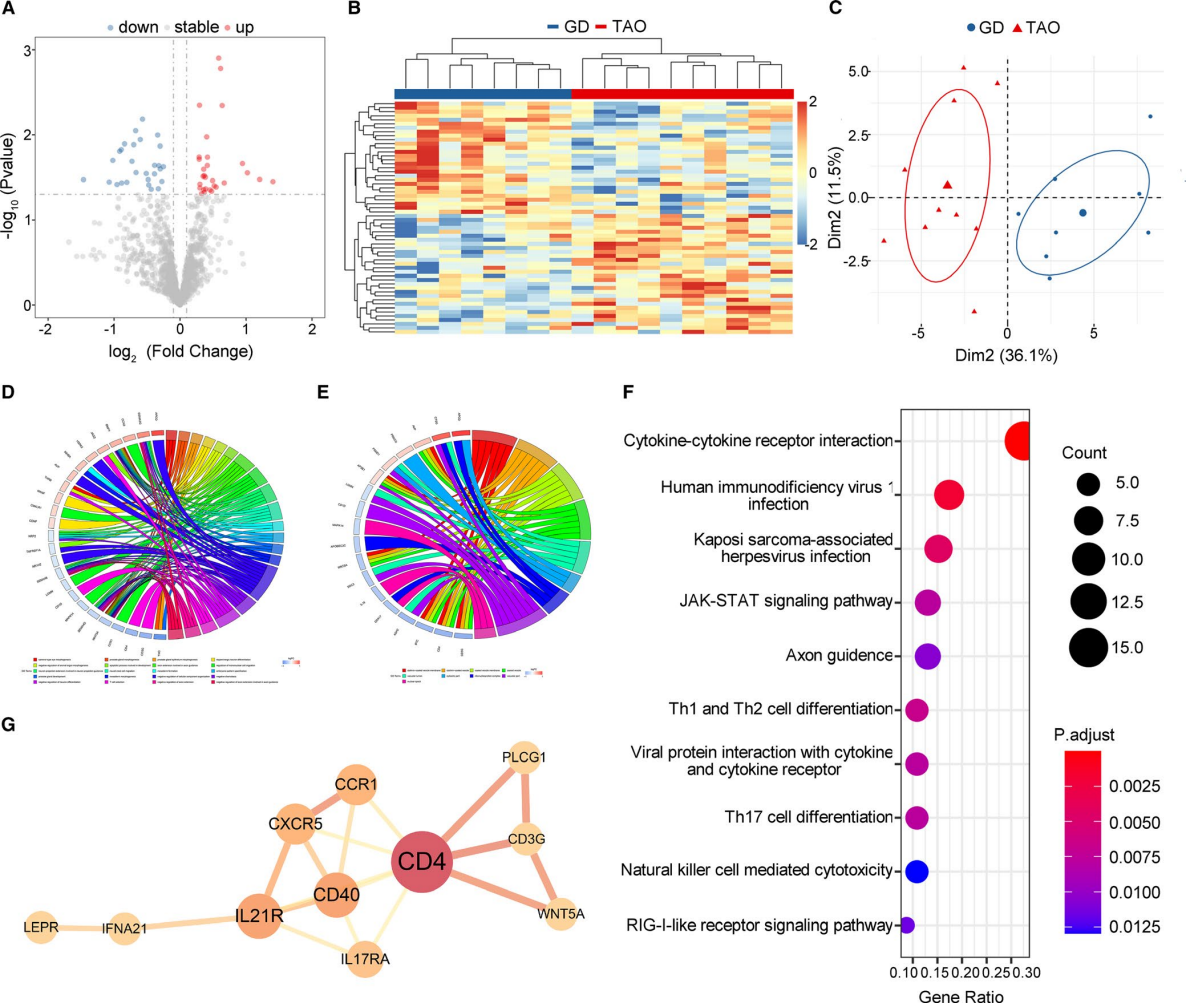
Identification of differentially expressed immune‐related genes (IRGs) in TAO vs GD set. (A) Volcano plot showed differentially expressed IRGs between TAO and GD group. Blue dots represented down‐regulated genes and red dots were up‐regulated genes. (B) Heatmap and (C) PCA plot showed obvious differences with those differentially expressed IRGs in TAO and GD group. (D) Gene Ontology (GO) enrichment analysis of differentially expressed IRGs among biological processes and (E) cellular components. Circos plots showed the relationship between genes and GO terms. (F) The top 10 most significant Kyoto Encyclopedia of Genes and Genomes pathways. (G) Protein‐protein interaction network of potential key IRGs. TAO, thyroid‐associated ophthalmopathy; GD, Graves’ disease; PCA, principal component analysis

The expression profile of IRGs in the TAO set in the GSE9340 data set (thyrocytes; TAO vs GD) was also analysed. A total of 935 DEGs were identified (Figure S1B). Among these genes, 58 were IRGs; 30 were up‐regulated and 28 were down‐regulated (Figure 3A). The heatmap and PCA plot showed that the differentially expressed IRGs can clearly distinguish the TAO and GD groups (Figure 3B,C). GO functional enrichment analysis results for the 58 IRGs showed that the most enriched terms were ‘regulation of mononuclear cell migration’ in the BP category, ‘clathrin‐coated vesicle membrane’ in the CC category and ‘hydrolase activity’ in the MF category (Figure 3D,E, Figure S2). Similarly, the cytokine‐cytokine receptor interaction pathway was the most enriched KEGG pathways (Figure 3F), and PPI network analysis identified 11 potential key IRGs in TAO (Figure 3G).

### 3.3 Venn diagram identification of the TAO‐specific key IRGs and pathways among GD‐related factors

**FIGURE 4:**
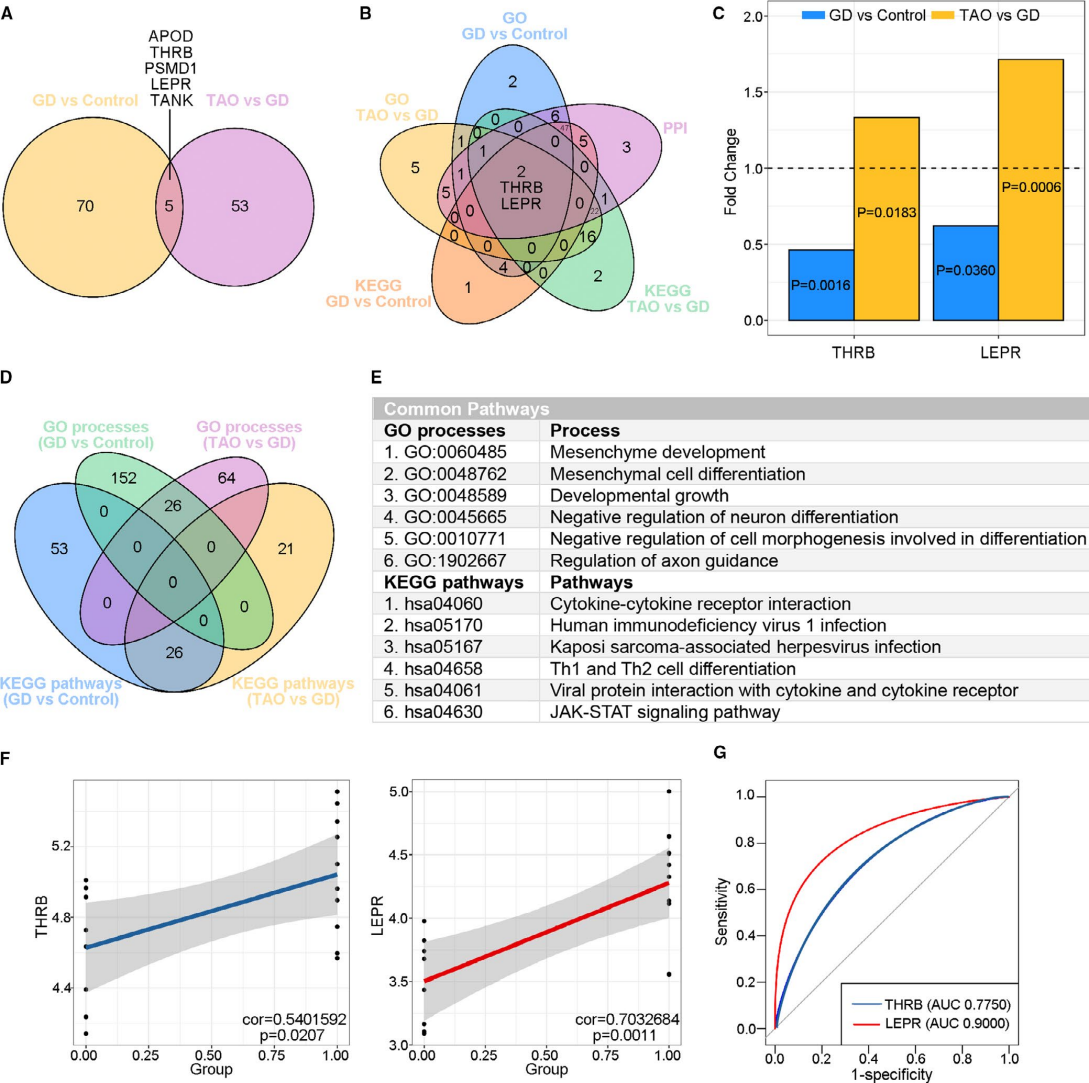
Identification of key immune‐related genes (IRGs) in TAO. (A) Venn diagram showed 75 and 58 differentially expressed IRGs in the GD vs HC set and TAO vs GD set, respectively. A total of 5 shared key IRGs were identified. (B) Five‐set Venn diagram showed a combination of all differentially expressed IRGs of Gene Ontology (GO) processes and Kyoto Encyclopedia of Genes and Genomes (KEGG) pathways in the GD vs HC set and TAO vs GD set, respectively. A total of 2 common IRGs were identified. (C) Fold change and P value of the 2 common IRGs in the GD vs HC set and TAO vs GD set. (D) Four‐way Venn diagram of GO processes and KEGG pathways detected in the GD vs HC set and TAO vs GD set. A total of 26 GO processes and 6 KEGG pathways were identified in common between the GD vs HC set and TAO vs GD set. (E) Details of the common pathways from Venn diagram analysis. (F) The correlations of the expression of the 2 common key IRGs with TAO state in TAO vs GD set by linear regression analysis. (G) ROC curves of the 2 common key IRGs for diagnosing TAO. ROC curves were considered significant with AUC value >0.8. GD, Graves' disease; HC, healthy control; TAO, thyroid‐associated ophthalmopathy; AUC, area under curve

A total of 5 potential TAO‐specific key IRGs were identified by Venn diagram analysis; these genes were detected based on the differentially expressed IRGs in the two sets (Figure 4A). Next, we utilized Venn diagram analysis to select the IRGs and pathways obtained via the abovementioned analyses (GO, KEGG and PPI analyses) of the GD and TAO sets, which were crucial for distinguishing the GD and TAO states. The results identified the thyroid hormone receptor β (*THRB*) and *LEPR* genes, both of which were among the 5 shared key genes from the analysis of differentially expressed IRGs (Figure 4B), indicating that they may be the best predictors or treatment targets for further validation in TAO. Both genes were down‐regulated in the GD set (*THRB*: fold change 0.46, *P* = .0016; *LEPR*: fold change 0.62, *P* = .0360) but up‐regulated in the TAO set (*THRB*: fold change 1.33, *P* = .0183; *LEPR*: fold change 1.71, *P* = .0006; Figure 4C). Additionally, 26 GO processes and 26 KEGG pathways (including cytokine‐cytokine receptor interaction and Th1 and Th2 cell differentiation) were shared by these two sets (Figure 4D,E, Appendix S1 and S2). Venn diagram analysis assisted us in detecting potential key IRGs and pathways in TAO pathogenesis.

### 3.4 Linear regression and ROC curve analysis of the two shared key IRGs

Subsequently, we conducted linear regression and ROC curve analyses of the two potential key IRGs (*THRB* and *LEPR*). In the TAO set, both *THRB* and *LEPR* genes were positively correlated with the TAO state (*THRB*: *P* = .0207, *LEPR*: *P* = .0011; Figure 4F). ROC curve analysis showed that the AUCs were 0.7750 and 0.900 for *THRB* and *LEPR*, respectively (Figure 4G). Considering that the AUC for *LEPR* was >0.8 but that for *THRB* was <0.8, we selected *LEPR* as the key gene involved in the immunopathogenesis of TAO as well as a potential early‐warning biomarker and interventional target to prevent TAO in GD patients.

### 3.5 GSEA: Robust immune response–related processes and pathways are prominent in the group of TAO patients with high *LEPR* expression

**FIGURE 5:**
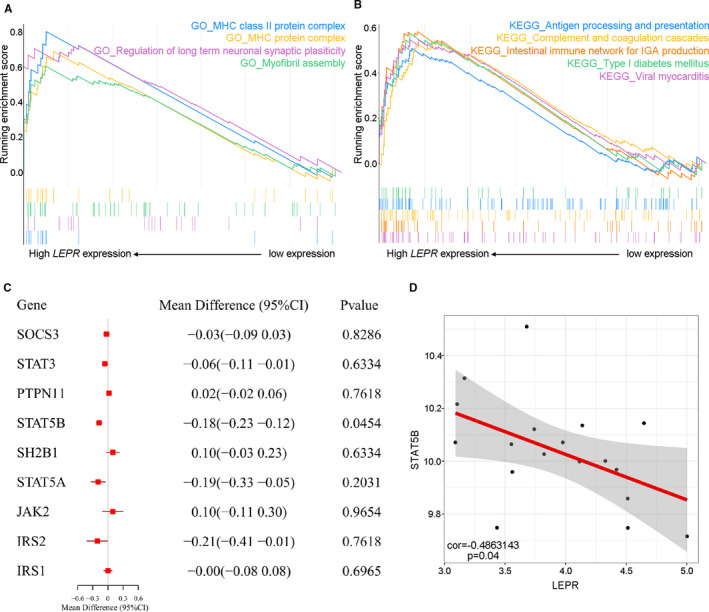
Molecular function and characteristics of *LEPR* in TAO. (A‐B) Gene set enrichment analysis (GSEA) showed (A) MHC protein complex and MHC class II protein complex in GO processes and (B) antigen processing and presentation pathway, complement and coagulation cascade pathway together with type 1 diabetes mellitus pathways among Kyoto Encyclopedia of Genes and Genomes (KEGG) pathways were obviously enriched in TAO patients with up‐regulated *LEPR* gene. (C) Forest plots of mean difference showed leptin signalling pathway–related gene differences between TAO and GD group. (D) The correlations of the expression of the differentially expressed genes in leptin signalling pathway with *LEPR* expression in TAO vs GD set. GD, Graves’ disease; HC, healthy control; TAO, thyroid‐associated ophthalmopathy

To determine the possible functional pathways affected by the *LEPR* gene in the TAO state, we performed GSEA to map the BP (Figure 5). In the group of TAO patients with high *LEPR* expression, antigen presentation–associated BP, including MHC protein complex and MHC class II protein complex, were up‐regulated (Figure 5A). Similarly, the KEGG pathways antigen processing and presentation pathway, complement and coagulation cascade pathway and type 1 diabetes mellitus pathway were obviously enriched in TAO patients with *LEPR* gene up‐regulation (Figure 5B). These results suggested that high *LEPR* expression on thyrocytes may contribute to the pathogenesis of TAO by up‐regulating the antigen presentation pathway.

### 3.6 Leptin signalling pathway–related genes in the TAO set

To investigate the molecular mechanism of the *LEPR* gene in the pathogenesis of TAO, we focused on the genes in the leptin signalling pathway that may be targets for *LEPR*. The gene list was obtained from the Reactome Pathway Database. The expression profiles of the 9 genes in the leptin signalling pathway in the TAO set are shown in Figure 5C. Among these genes, the expression level of the signal transducers and activators of transcription 5B (*STAT5B*) gene was significantly reduced in the TAO set (.0454) and was correlated with the *LEPR* expression level (*STAT5B*: *P* = .04; Figure 5D). Thus, *STAT5B* was the potential target molecule of *LEPR* involved in the TAO pathogenesis in thyrocytes.

### 3.7 *LEPR* expression in orbital tissues from TAO patients

**FIGURE 6:**
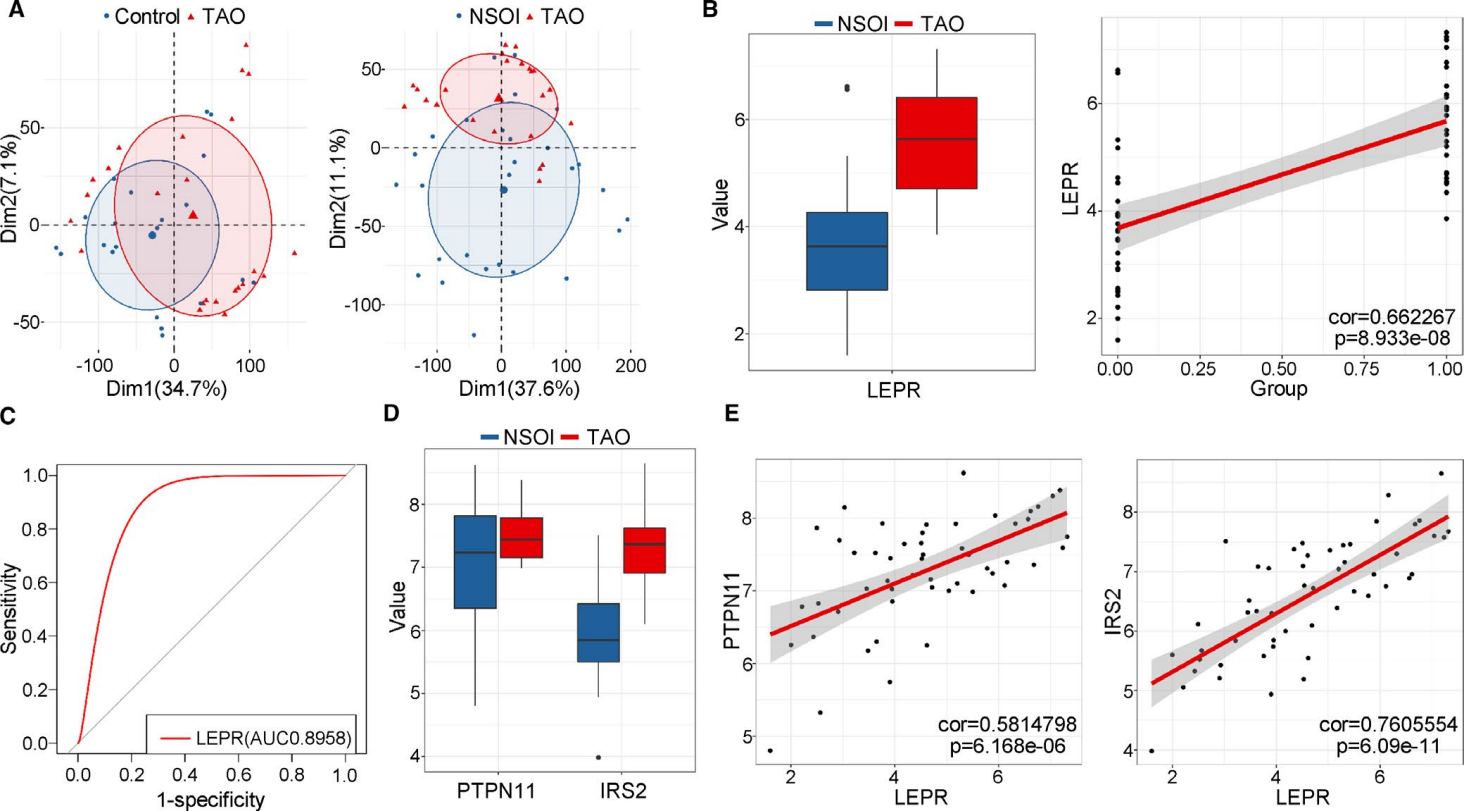
*LEPR* expression in orbital tissues from TAO patients. (A) PCA plot showed differences with all expressed genes between TAO and (left) HC (right) NSOI. The overlap was more in the TAO vs HC set compared with the TAO vs NSOI group. (B; left) Boxplot showed the expression of *LEPR* orbital tissues from TAO and NSOI group. Blue represented NSOI and red for TAO. Error bars showed SEM. (right) The correlations of the expression of *LEPR* with TAO state in TAO vs NSOI set by linear regression analysis. (C) ROC curve of the *LEPR* for diagnosing TAO state in TAO vs NSOI set. (D) Boxplot showed the expression of *PTPN11* and *IRS2* (leptin signalling pathway–related genes) in TAO vs NSOI set. Blue represented NSOI and red for TAO. Error bars showed SEM. (E) The correlations of the expression of (left) *PTPN11* and (right) *IRS2* with *LEPR* expression in TAO vs NSOI set

The GSE58331 data set included orbital tissues from TAO patients, NSOI patients and HCs. Because the PCA plots indicated that the discrimination between samples from TAO patients and those from HCs was not as good as that between samples from patients with TAO and samples from NSOI patients (Figure 6A), we considered the NSOI group as a control group. Consistent with previous results in thyrocytes from TAO patients, *LEPR* was obviously increased in orbital tissues from TAO patients compared with those from NSOI patients (*P* = 6.049e‐08; Figure 6B). In addition, the *LEPR* gene was positively correlated with the TAO state (*P* = 8.933e‐08; Figure 6B), and the ROC curve showed that *LEPR* expression can distinguish patients with TAO from controls (AUC: 0.8958; Figure 6C). In addition, the levels of *PTPN11* and *IRS2* in the leptin signalling pathway were significantly increased (*P* =.03 and 6.591e‐09; Figure 6D), and the expression of both of these genes was positively correlated with *LEPR* expression (*P* = 6.168e‐06 and 6.09e‐11; Figure 6E).

### 3.8 Potential function of *LEPR* in orbital tissues from TAO patients

**FIGURE 7:**
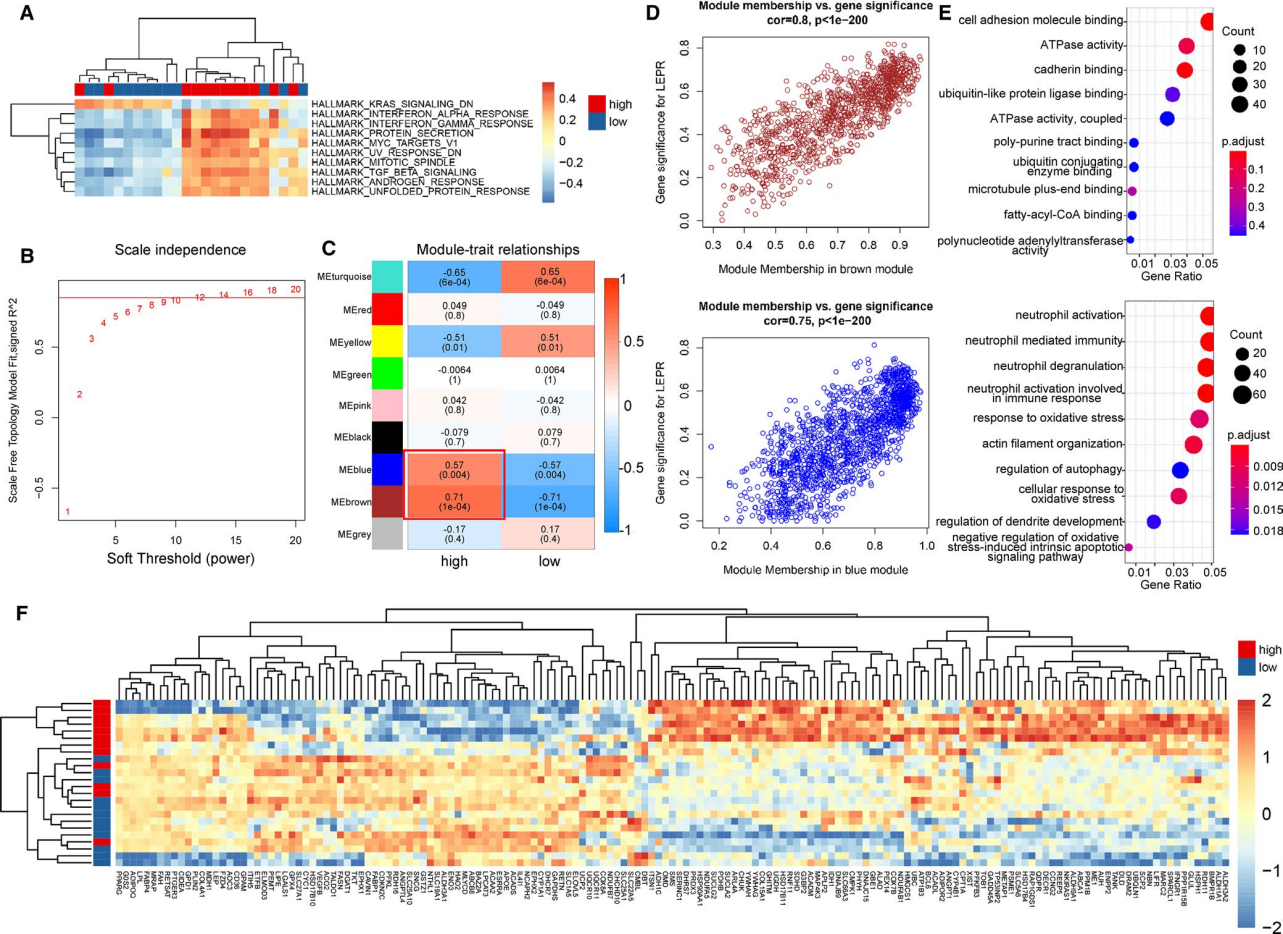
*LEPR* expression in orbital tissues from TAO patients. (A) Heatmap showed gene set variable analysis (GSVA) results that protein secretion, interferon alpha and gamma response were enriched in hallmark set among TAO patients with high *LEPR* expression. Blue represented NSOI and red for TAO. (B) Analysis of the scale‐free fit index for various soft‐thresholding powers. (C) Heatmap between the correlation between modules and high or low *LEPR* expression (each cell contained the correlation coefficient and corresponding *P* value). (D)The gene significance for *LEPR* expression in the (upper) brown and (bottom) blue module (one dot represented one gene in the yellow module). (E) Kyoto Encyclopedia of Genes and Genomes (KEGG) enrichment analysis of (upper) brown and (bottom) blue module genes. (F) Heatmap showed expression profile of genes with top 50% mad value in ‘adipogenesis’ and ‘fatty acid metabolism’ from hallmark set. HC, healthy control; TAO, thyroid‐associated ophthalmopathy; NSOI, non‐specific orbital inflammation; PCA, principal component analysis

To better understand the function of *LEPR* in orbital tissues from TAO patients, we conducted hallmark, KEGG and BIOCARTA pathway analyses for the DEGs in the groups of TAO patients with high and low *LEPR* expression via GSVA. Protein secretion and the interferon alpha and gamma response were enriched in TAO patients with high *LEPR* expression (Figure 7A). Among TAO patients with high *LEPR* expression, the most highly enriched pathways identified in the KEGG and BIOCARTA pathway analyses were protein export and LYM pathway, respectively (Figure S3A,B).

In addition, through WGCNA analysis of genes with the top 75% median absolute deviation (mad) values in TAO patients (with 0.85 set as the correlation coefficient threshold and 12 as the soft‐thresholding power, Figure 7B), 8 co‐expression modules were constructed (Figure S4). Module‐trait correlation analyses showed that multiple modules were positively related to increased expression of *LEPR* (Figure 7C). Among these modules, the brown and blue modules were the most significantly associated with high *LEPR* expression in TAO patients (brown: *P* < 1e‐04, blue: *P* = .004; Figure 7D; Appendix S3 and S4). GO analysis showed that the genes in brown and blue module were the most highly enriched in the MF term ‘cell adhesion molecule binding’ and the BP term ‘neutrophil activation’, consistent with the GSVA results for BIOCARTA pathways (Figure 7E). In addition, considering that *LEPR* was related to lipid metabolism, we explored the expression profile of genes with the top 50% mad values in the ‘adipogenesis’ and ‘fatty acid metabolism’ hallmark gene sets. The results suggested that the expression of lipid metabolism–related genes can discriminate TAO patients with high *LEPR* expression from those with low *LEPR* expression and that genes such as *ACADM*, *COL15A1* and *ADH1C* are up‐regulated in TAO patients with high *LEPR* expression. These findings implied that *LEPR* participates in TAO progression via the lipid metabolism pathway (Figure 7F).

## 4 DISCUSSION

In this study, we screened two public microarray data sets containing thyroid samples (E‐MEXP‐2612 and GSE9340) to investigate key IRGs in the processes of TAO and used the GSE58331 data set, containing orbital tissues from TAO patients, to validate our findings. We firstly found that *LEPR* is not only a key gene involved in the immunopathogenesis of TAO but also a diagnostic biomarker for TAO among GD patients. In addition, bioinformatic analyses were conducted to explore the potential functional processes and potential targets of *LEPR*.

*LEPR*, the gene encoding the leptin receptor, belongs to the class I cytokine receptor superfamily, and the long isoform of *LEPR* transmits extracellular leptin signals via the Janus kinase (JAK) and STAT signalling pathways.^26^ In addition to its role in conveying information about regulating energy homeostasis, leptin signalling is also a crucial participant in the immune system.^26^, ^27^ *LEPR* deficiency in CD4+ T cells has been confirmed to reduce the capacity for differentiation towards a Th17 phenotype by down‐regulating the activation of *STAT3* and its downstream targets.^28^ Additionally, *LEPR* expression is increased in both mouse and human macrophages, while *LEPR* ablation in macrophages diminished inflammation both in vitro and in vivo by augmenting lysosomal function.^29^ In addition, *LEPR* contributes to the pathogenesis of several autoimmune diseases, including specific clinical phenotypes (pericarditis and photosensitivity) of systemic lupus erythematosus (SLE) and rheumatoid arthritis (RA).^30^, ^31^ Above all, *LEPR* plays a significant role in both the adaptive and innate immune responses and is closely associated with autoimmune diseases.

Similar to its expression pattern in other autoimmune diseases mentioned above, *LEPR* was up‐regulated in thyrocytes from patients with TAO, and TAO patients with high *LEPR* expression showed more intensive antigen processing and presentation processes than those with low *LEPR* expression. Mechanistically, due to increased TSHR auto‐antigen presentation, high levels of anti‐TSHR antibodies recognize TSHR on orbital fibroblasts and, in cooperation with interferon gamma and tumour necrosis factor (TNF), lead to the autoimmune characteristic of TAO.^3^ Moreover, the antigen presentation‐related *HLA‐DR14* and *DQ1* genes may be genetic markers of a predisposition to the development of severe TAO,^32^ further suggesting the correlation between the antigen presentation pathway and TAO as well as the crucial role of *LEPR* in the progression of TAO.

Furthermore, we detected an increase in *LEPR* expression in orbital tissues from TAO patients, which corresponds to findings from previous studies that showed increased leptin expression in orbital tissues from patients with TAO and that adipocytes derived from orbital preadipocyte fibroblasts stimulated with TSHR can produce 6‐37 times more leptin than those from controls.^33^, ^34^ The main pathological alteration in orbital tissues from TAO appears to involve both the extraocular muscles and the orbital fat compartments.^35^ The results of hallmark and KEGG analyses via GSVA in TAO patients with high *LEPR* expression suggest that *LEPR* is involved in protein secretion. Enlarged extraocular muscles from patients with TAO are widely separated by an amorphous accumulation of granular material consisting primarily of collagen fibrils and glycosaminoglycans.^36^ Thus, it is reasonable to infer that high *LEPR* expression in orbital tissues may promote the production of collagen fibrils, which can further induce muscle body oedema and exacerbate the TAO state.^37^ The other pathological alteration in extraocular muscles is diffuse and focal immune infiltration.^35^ Similarly, the results of GSVA of BIOCARTA pathways and WGCNA suggested that adhesion molecules participate in the progression of TAO with increased *LEPR* expression, which has been confirmed to be correlated with the progression of TAO and subsequently induces immune cell infiltration.^38^, ^39^, ^40^ In summary, *LEPR* presumably participates in the enlargement of extraocular muscles during TAO progression by promoting collagen fibril production and immune cell infiltration.

Considering that orbital fat compartments were also involved in TAO and that *LEPR* was related to lipid metabolism, we explored the correlation between these factors in TAO and found that genes in the lipid metabolism pathway could discriminate TAO patients with high *LEPR* expression from those with low *LEPR* expression. Previous results have revealed increased mRNA expression of the adipocyte‐specific genes leptin, adiponectin, fatty acid synthase, adipocyte fatty acid binding protein (AP2) and PPAR‐γ in TAO‐affected adipose tissue, implying that de novo adipogenesis occurs in orbital tissues in TAO.^35^ Additionally, non‐targeted metabolite profiling of blood from TAO patients exhibited different profiles in the GD group and HC group, and the combination of proline and 1,5‐anhydroglucitol was identified as a GO‐specific modulator.^41^ Moreover, plasma cholesterol is considered a risk factor for TAO, and a significant relationship was found between the therapeutic efficacy of intravenous methylprednisolone and pre‐treatment triglyceride levels in TAO patients.^42^, ^43^ Taken together, these findings indicate that *LEPR* may contribute to the adipogenesis and lipid metabolism alterations observed in patients with TAO and may even result in the unsatisfactory treatment effects of glucocorticoids.

However, the current study has limitations. On the one hand, further research concerning the exact mechanism of the *LEPR* gene in TAO was not performed. On the other hand, the three data sets that we analysed utilized distinct platforms for gene expression analysis and were assembled from quite different populations. Therefore, *LEPR* expression still needs to be explored in TAO patients of more different races.

In summary, our study revealed that *LEPR* is a key gene in the immunopathogenesis of TAO and that its up‐regulation promotes antigen presentation by thyrocytes and mediates protein secretion, immune cell adhesion and adipogenesis pathway activity in orbital tissues. These findings provide new insights into diagnostic and therapeutic biomarkers for TAO, although the specific molecular mechanism and functional pathway of *LEPR* in TAO needs further exploration.

## CONFLICT OF INTEREST

The authors confirm that there are no conflicts of interest.

## AUTHOR CONTRIBUTION

**Ziyi Chen:** Conceptualization (lead); Data curation (lead); Investigation (lead). **Zhe Chen:** Data curation (equal); Investigation (equal). **Jingya Wang:** Data curation (equal); Methodology (equal). **Meng Zhang:** Data curation (equal). **Xiaofei Wang:** Methodology (equal). **Deji Cuomu:** Data curation (equal). **Bingyin Shi:** Funding acquisition (equal); Supervision (equal); Validation (equal); Writing – review and editing (equal). **Yue Wang:** Conceptualization (lead); Funding acquisition (lead); Supervision (lead).

## Supporting information

Fig S1

Fig S2

Fig S3

Fig S4

Appendix S1

Appendix S2

Appendix S3

Appendix S4

## Data Availability

The data sets used and/or analysed during the current study are available from the corresponding author on reasonable request. The public data source: GEO database (https://www.ncbi.nlm.nih.gov/geo/) and ArrayExpress database (https://www.ebi.ac.uk/arrayexpress/).
