# Supplementary figures and images for: Leptin receptor is a key gene involved in the immunopathogenesis of thyroid‐associated ophthalmopathy
Source: J Cell Mol Med. 2021 May 14;25(12):5799–810. doi: 10.1111/jcmm.16605 (PMC8184729; doi:10.1111/jcmm.16605)

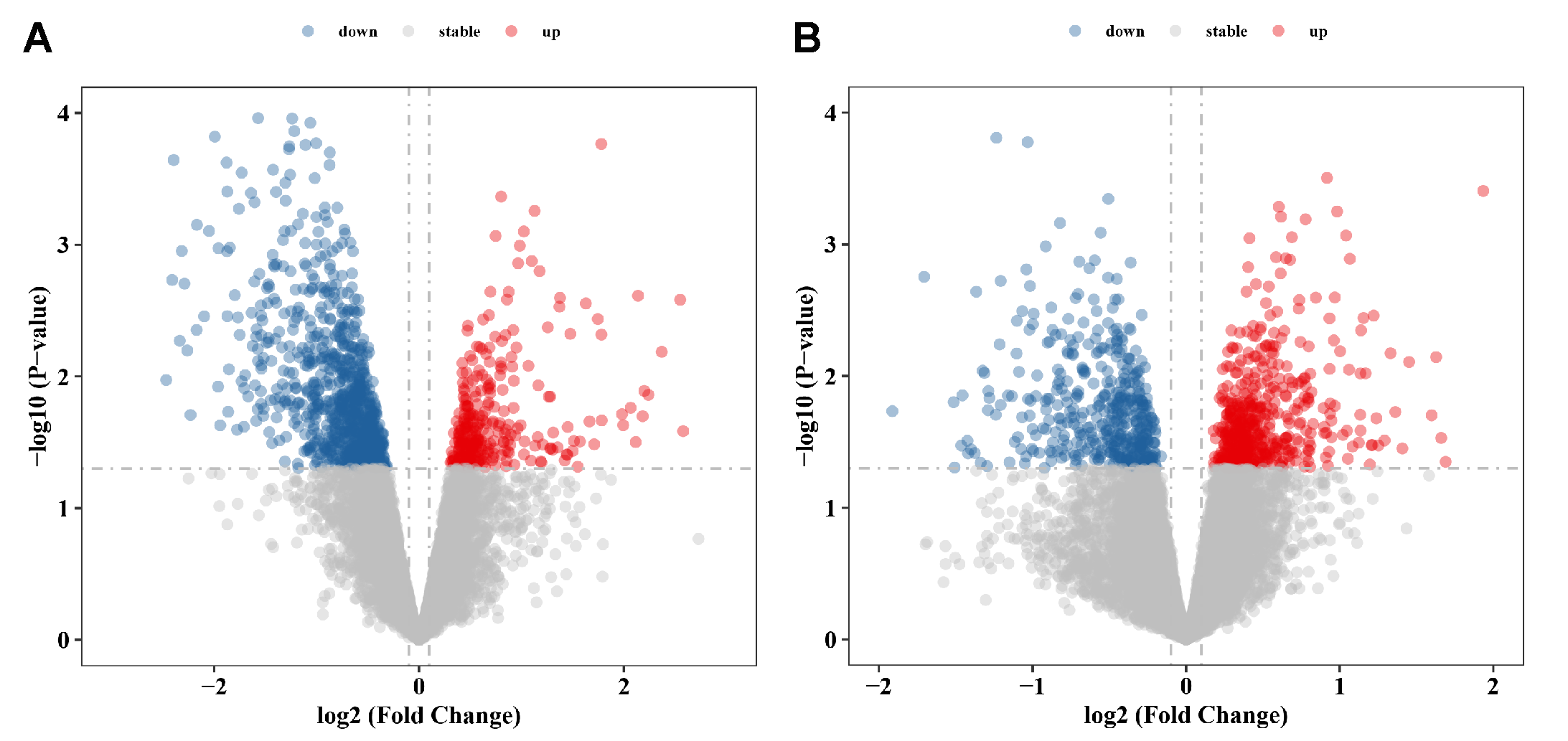

Supplement: Supplementary file 1 — Fig S1 [file JCMM-25-5799-s001.tif]

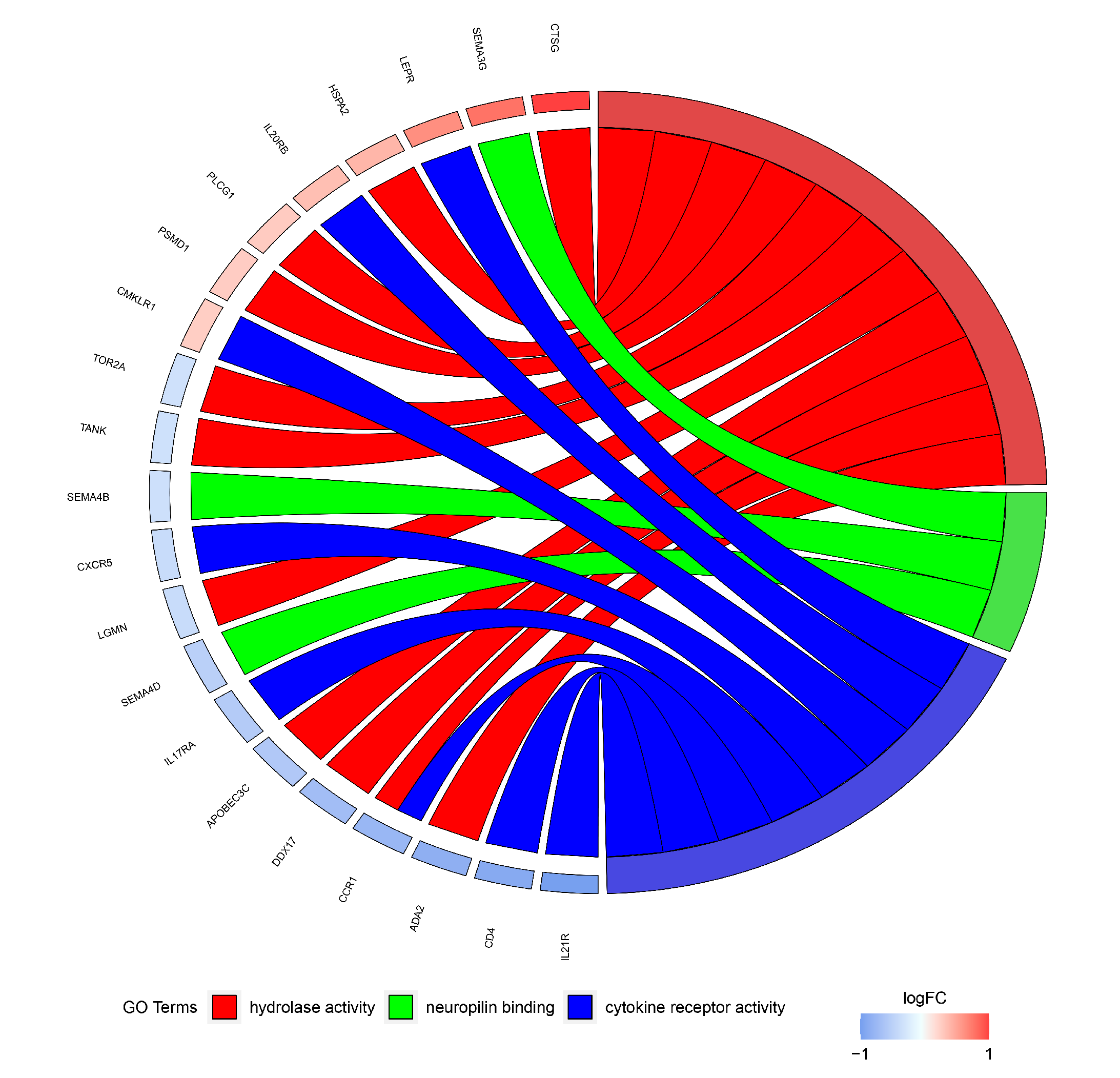

Supplement: Supplementary file 2 — Fig S2 [file JCMM-25-5799-s004.tif]

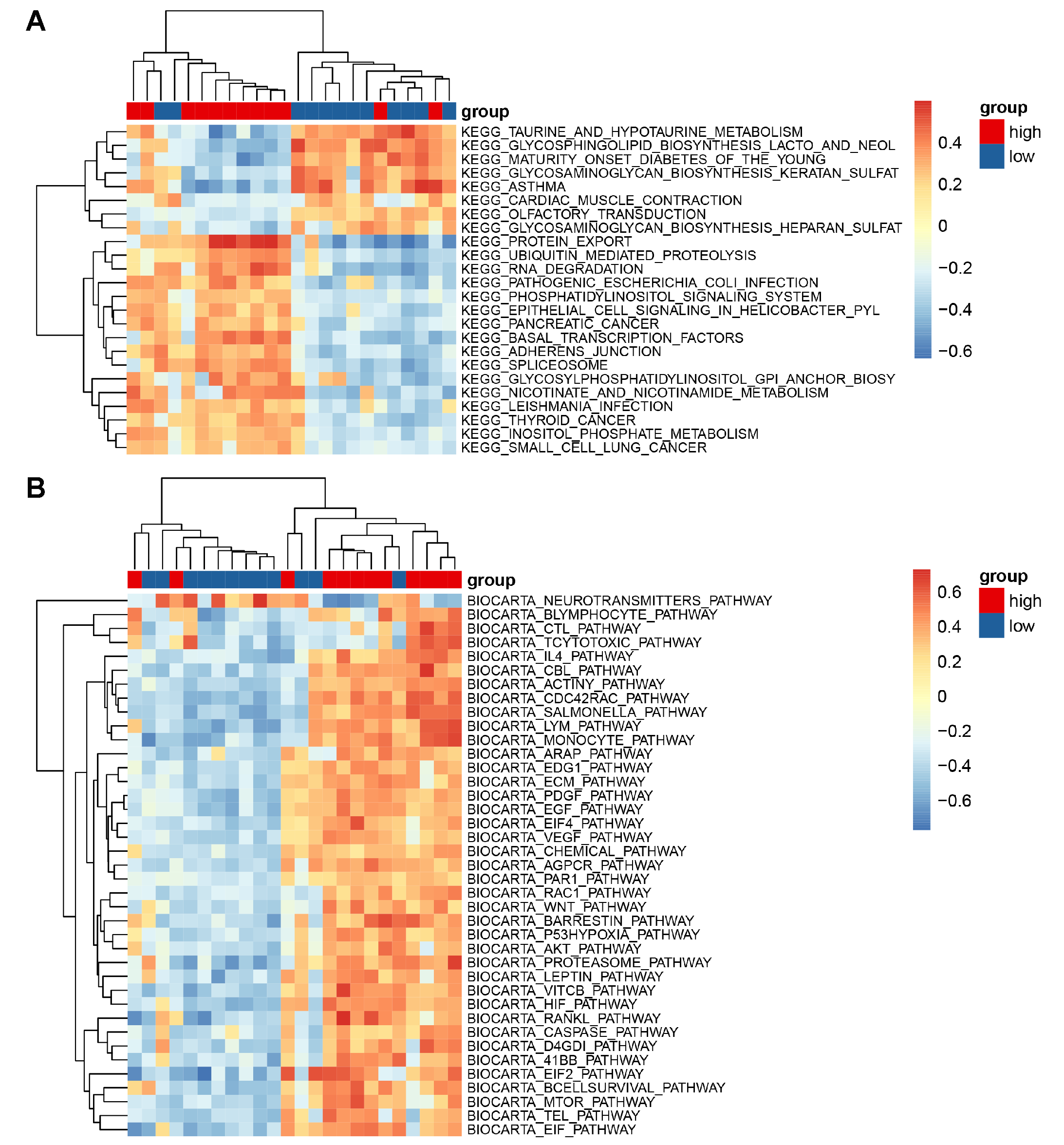

Supplement: Supplementary file 3 — Fig S3 [file JCMM-25-5799-s005.tif]

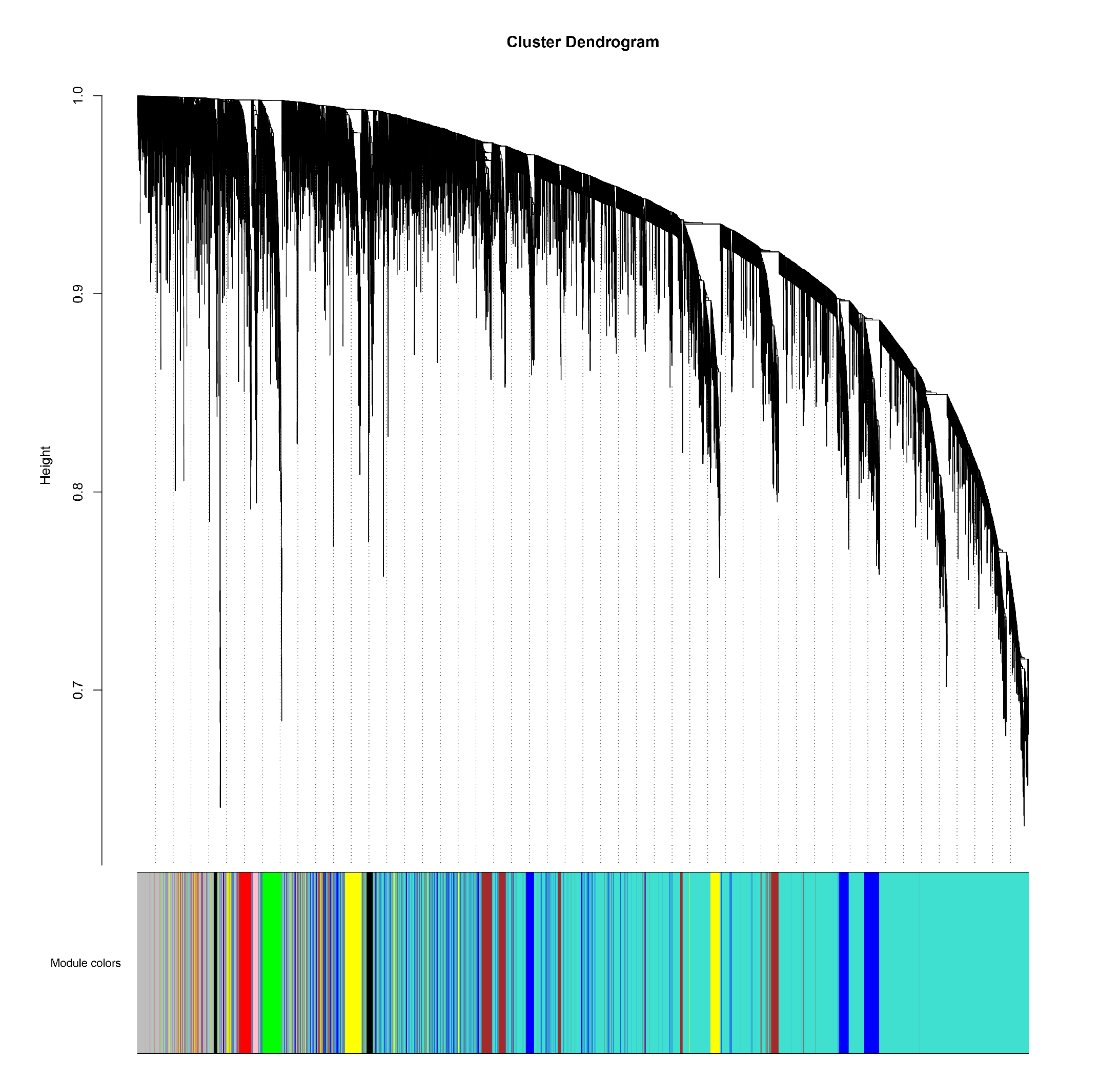

Supplement: Supplementary file 4 — Fig S4 [file JCMM-25-5799-s003.tif]
